# Editorial: Supramolecular Chirogenesis in Chemical and Related Sciences

**DOI:** 10.3389/fchem.2021.679332

**Published:** 2021-04-06

**Authors:** Yue Sun, Riina Aav, Akihiko Tsuda, Hiroyuki Miyake, Keiji Hirose, Victor Borovkov

**Affiliations:** ^1^Hubei Key Laboratory of Catalysis and Materials Science, College of Chemistry and Material Sciences, South-Central University for Nationalities, Wuhan, China; ^2^Department of Chemistry and Biotechnology, School of Science, Tallinn University of Technology, Tallinn, Estonia; ^3^Department of Chemistry, Graduate School of Science, Kobe University, Kobe, Japan; ^4^Department of Chemistry, Graduate School of Science, Osaka City University, Osaka, Japan; ^5^Department of Materials Engineering Science, Graduate School of Engineering Science, Osaka University, Toyonaka, Japan

**Keywords:** supramolecular chemistry, chirogenesis, porphyrins, self-assembly, circular dichroism, chirality, circularly polarized luminescence

Chirality being a phenomenon of describing the ability of any object to exist as a pair of non-superimposable mirror images has aroused extensive attention as it is directly correlated with numerous biological systems (e.g., transcription, storage, processing of genetic information, and functioning living organisms). Specific to all living organisms, the mentioned microscopic self-assembled nanostructures exhibiting the supramolecular chirogenesis are spontaneously arranged from chiral building blocks through non-covalent interactions or from achiral components being influenced by an external chiral field (Hembury et al., 2008; Liu et al., 2015; Sun et al., 2018a,b). A typical example refers to the conformations of proteins with different chirogenic arrangement (e.g., α-helix, β-sheet, and tertiary structure). An in-depth study on the chirogenesis phenomenon will present more insights into the self-assembly behavior in biological systems, as well as will help expand the application in materials science (Sun et al., 2016).

While the term of supramolecular chirogenesis (Scheme 1) was firstly introduced in 2000 (Borovkov et al., 2000), in general, this effect can be generated in two major forms (Morris and Bu, 2010; Zhang et al., 2012). On one hand, chirogenesis contains chiral molecular components and mixed chiral/achiral molecules (Goh et al., 2010; Ie et al., 2015; Nakakoji et al., 2020). On the other hand, it can be arisen from achiral molecules as impacted by the spontaneous symmetry breaking when the self-assembled systems are being formed. Alternatively, it can be also generated from achiral components, which are able to assembly into a chiral system upon external chiral influence. This Research Topic collected 14 original articles from 85 authors on supramolecular chirogenesis in chemical and relevant sciences.

**Scheme 1:**
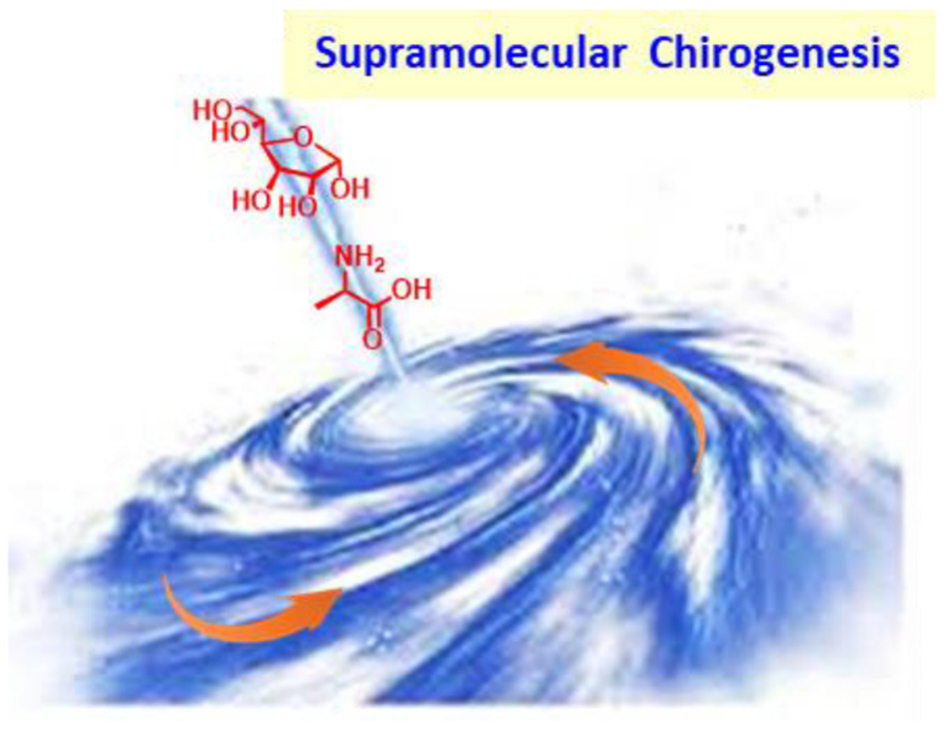
Schematic representation of molecular chirality and supramolecular chirogenesis (Borovkov et al., 2000).

Porphyrins refer to be prominent host molecules, which can be commonly used to study chiral supramolecular systems for their unique planar structures and abundant chemical property. In this respect, Stefanelli et al. reported that porphyrin appended with l- or d-prolinate is capable of generating remarkable supramolecular chirality, emerging from the stereospecific proline-appended group. Also, Lu et al. prepared corresponding porphyrin dimers linked with 2,2′-diamino-1,1′-binaphthyl. The porphyrin dimers are able to form the 1:1 sandwich host-guest complexes with chiral diamines at low concentrations. It is noteworthy that the authors could monitor the process of intermolecular chirality attributed to the existence of short chiral linkage units by using circular dichroism (CD) spectroscopy. Furthermore, Gaeta et al. disclosed that chiral porphyrin hetero-aggregates can act as templates to generate the chiral structures during the oxidative conversion of amino acid.

Besides the chirogenic porphyrin chemistry, Fujiki et al. demonstrated that the signal of CPL (circularly polarized luminescence) and CD appeared through the intermolecular chirality transfer upon lanthanide (Eu^III^ and Tb^III^) tris(β-diketonate) exposed to poly- and monosaccharide alkyl esters and α-pinene. Also, Nakakoji et al. investigated the ability of chiral discrimination between copper(II)-chiral tetradentate ligand and a chiral amino acid with the isotopically labeled/unlabeled enantiomer method. As revealed from this study, the matching steric interaction accounts for the observed chiral recognition. Trapp described that a transient stereodynamic catalyst is capable of efficiently amplifying the Soai's asymmetric autocatalysis. The coordination bond provides a good platform to study the property of materials (Chen et al., 2021; Li et al., 2021). Nishinaka et al. indicated that a novel coordination-driven based polymer composed of phenylene-bridged bipyrrole and palladium(II) ion achieved the π-electronic communications and exciton coupling in the absorption spectrum.

Numerous fields generated the interest in macrocycles exhibiting the optically-active characteristics (Aav and Mishra, 2018). Okada et al. summarized the synthesis and property of the optically-active phthalocyanines, as well as their related azamacrocycles from 2010 to 2020, which are of high significance for chemists to design highly desirable chiral macrocycles from achiral molecules. As summarized by Qiu et al., the C3 symmetrical cages were employed as efficient scaffolds to study chiral dynamics. With the use of non-linear chiroptical techniques, the chirality at surfaces can be clarified from novel perspectives. Gogoi et al. reviewed that chemists adopted the linear and non-linear optical methods to quantify the surface chirality, as an attempt to solve vital issues in surface biochemistry.

Catalytic asymmetric chemistry is another field of supramolecular chirogenesis. In this respect, halogen bonding, with its high directionality, has been recognized as an attractive interaction for new molecular assemblies and its use in chiral systems just started to emerge. In this respect, Kaasik and Kanger demonstrated the use of halogen bonding in catalytic stereoselective processes. Also, Kananovich et al. highlighted recent achievements in the catalytic enantioselective oxidations utilizing molecular oxygen, which placed the stress on the mechanisms of dioxygen activation and chirogenesis in the mentioned chemical transformations.

In related development Siligardi et al. outlooked that researchers used the CD imaging at high spatial resolution at Diamond B23 beamline to determine the homogeneity of the supramolecular structures of thin films deposited on fused quartz substrates.

In summary, this Research Topic collected various aspects of the supramolecular chirogenesis in chemical and related sciences. To be specific, the topic illustrates the emergence and characterization of chirogenesis and its potential applications. It is expected that this topic will guide the design and functionality of various chiral structures. The field of supramolecular chirogenesis and relevant sciences are significantly promising in understanding the origins of life in nature, as well as in developing novel medicines and smart materials.

## Author Contributions

All authors listed have made a substantial, direct and intellectual contribution to the work, and approved it for publication.

## Conflict of Interest

The authors declare that the research was conducted in the absence of any commercial or financial relationships that could be construed as a potential conflict of interest.

## References

[B1] Aav R. Mishra K. A. (2018). The breaking of symmetry leads to chirality in cucurbituril-type hosts. Symmetry 10:98. 10.3390/sym10040098

[B2] Borovkov V. V. Lintuluoto J. M. Inoue Y. (2000). Supramolecular chirogenesis in bis(zinc porphyrin): an absolute configuration probe highly sensitive to guest structure. Org. Lett. 2, 1565–1568. 10.1021/ol0000556 10841480

[B3] Chen Z. Sun Y. Li H. (2021). Fabrication of subnanochannels by metal–organic frameworks. Matter 4, 772–774. 10.1016/j.matt.2021.02.004

[B4] Goh M. Matsushita S. Akagi K. (2010). From helical polyacetylene to helical graphite: synthesis in the chiral nematic liquid crystal field and morphology-retaining carbonisation. Chem. Soc. Rev. 39, 2466–2476. 10.1039/b907990b 20571671

[B5] Hembury G. A. Borovkov V. V. Inoue Y. (2008). Chirality-sensing supramolecular systems. Chem. Rev. 108, 1–73. 10.1021/cr050005k 18095713

[B6] Ie M. Setsune J. Eda K. Tsuda A. (2015). Chiroptical sensing of oligonucleotides with a cyclic octapyrrole. Org. Chem. Front. 2, 29–33. 10.1039/C4QO00268G

[B7] Li R. H. Feng X. Y. Zhou J. Yi F. Zhou Z. Q. Men D.. (2021). Rhomboidal Pt(II) metallacycle-based hybrid viral nanoparticles for cell imaging. Inorg. Chem. 60, 431–437. 10.1021/acs.inorgchem.0c03095 33320662

[B8] Liu M. Zhang L. Wang T. (2015). Supramolecular chirality in self-assembled systems. Chem. Rev. 115, 7304–7397. 10.1021/cr500671p 26189453

[B9] Morris R. E. Bu X. (2010). Induction of chiral porous solids containing only achiral building blocks. Nat. Chem. 2, 353–361. 10.1038/nchem.628 20414234

[B10] Nakakoji T. Sato H. Ono D. Miyake H. Shinoda S. Tsukube H.. (2020). Mass spectrometric detection of enantioselectivity in three-component complexation, copper(II)-chiral tetradentate ligand-free amino acid in solution. Chem. Commun. 56, 54–57. 10.1039/C9CC07231D 31710051

[B11] Sun Y. Li S. Zhou Z. Saha M. L. Datta S. Zhang M.. (2018b). Alanine-based chiral metallogels via supramolecular coordination complex platforms: metallogelation induced chirality transfer. J. Am. Chem. Soc. 140, 3257–3263. 10.1021/jacs.7b10769 29290113 PMC5842145

[B12] Sun Y. Mei Y. Quan J. Xiao X. Zhang L. Tian D.. (2016). The macroscopic wettable surface: fabricated by calix[4]arene-based host–guest interaction and chiral discrimination of glucose. Chem. Commun. 52, 14416–14418. 10.1039/C6CC07956C 27901133

[B13] Sun Y. Zhang F. Quan J. Zhu F. Hong W. Ma J.. (2018a). A biomimetic chiral-driven ionic gate constructed by pillar[6]arene-based host–guest systems. Nat. Commun. 9, 2617. 10.1038/s41467-018-05103-w 29976986 PMC6033921

[B14] Zhang M. Qing G. Sun T. (2012). Chiral biointerface materials. Chem. Soc. Rev. 41, 1972–1984. 10.1039/C1CS15209B 22138816

